# Integrated transcriptomic and proteomic analysis identifies protein kinase CK2 as a key signaling node in an inflammatory cytokine network in ovarian cancer cells

**DOI:** 10.18632/oncotarget.7255

**Published:** 2016-02-08

**Authors:** Hagen Kulbe, Francesco Iorio, Probir Chakravarty, Carla S. Milagre, Robert Moore, Richard G. Thompson, Gemma Everitt, Monica Canosa, Alexander Montoya, Denis Drygin, Ioana Braicu, Jalid Sehouli, Julio Saez-Rodriguez, Pedro R. Cutillas, Frances R. Balkwill

**Affiliations:** ^1^ Centre for Cancer and Inflammation, Barts Cancer Institute, Queen Mary University of London, London, UK; ^2^ Centre for Haemato-Oncology, Barts Cancer Institute, Queen Mary University of London, London, UK; ^3^ Bioinformatics Core, The Francis Crick Institute, London, UK; ^4^ European Molecular Biology Laboratory – European Bioinformatics Institute, EMBL-EBI, Cambridge, UK; ^5^ Pimera, Inc., San Diego, CA, USA; ^6^ Cancer Genome Project, Wellcome Trust Sanger Institute, Cambridge, UK; ^7^ Tumorbank Ovarian Cancer Network, Department of Gynecology, Charité Universitätsmedizin Berlin, Berlin, Germany; ^8^ Department of Gynecology, Charité Universitätsmedizin Berlin, Berlin, Germany

**Keywords:** inflammatory cytokine networks, ovarian cancer, microenvironment, systems biology, therapeutics

## Abstract

We previously showed how key pathways in cancer-related inflammation and Notch signaling are part of an autocrine malignant cell network in ovarian cancer. This network, which we named the “TNF network”, has paracrine actions within the tumor microenvironment, influencing angiogenesis and the immune cell infiltrate.

The aim of this study was to identify critical regulators in the signaling pathways of the TNF network in ovarian cancer cells that might be therapeutic targets. To achieve our aim, we used a systems biology approach, combining data from phospho-proteomic mass spectrometry and gene expression array analysis. Among the potential therapeutic kinase targets identified was the protein kinase Casein kinase II (CK2).

Knockdown of CK2 expression in malignant cells by siRNA or treatment with the specific CK2 inhibitor CX-4945 significantly decreased Notch signaling and reduced constitutive cytokine release in ovarian cancer cell lines that expressed the TNF network as well as malignant cells isolated from high grade serous ovarian cancer ascites. The expression of the same cytokines was also inhibited after treatment with CX-4945 in a 3D organotypic model. CK2 inhibition was associated with concomitant inhibition of proliferative activity, reduced angiogenesis and experimental peritoneal ovarian tumor growth.

In conclusion, we have identified kinases, particularly CK2, associated with the TNF network that may play a central role in sustaining the cytokine network and/or mediating its effects in ovarian cancer.

## INTRODUCTION

Malignant cells generate complex networks of constitutively-produced chemokines, their receptors, growth factors and inflammatory cytokines, often as a consequence of oncogenic mutations [1-5]. These networks have paracrine actions in the tumor microenvironment impacting on tumor progression and spread [6].

Using preclinical models of ovarian cancer, *in silico* analyses of gene expression microarray datasets from over 500 patient samples and human ovarian cancer biopsies, we identified a malignant cell-autonomous cytokine network, which includes the stromal cell-derived factor CXCL12 and its receptor CXCR4, the inflammatory cytokines TNF, IL6, and vascular endothelial growth factor (VEGF) [7, 8]. Furthermore, we have demonstrated how autocrine CXCL12/CXCR4 signaling sustains this cytokine network through induction of TNF and that key pathways in cancer-related inflammation and NOTCH signaling appear to be part of this autocrine malignant cell network [2, 7, 8]. We have shown how this network promotes the malignant phenotype through paracrine actions on angiogenesis, the stromal signature and the immune cell infiltrate in both murine xenograft models and ovarian cancer, with an impact on overall survival [2, 9-12].

The dependency of network genes on TNF was demonstrated by their down-regulation in tumour cells from patients with advanced ovarian cancer following the infusion of anti-TNF antibodies [2, 13]. Therefore, we named this observation ‘the TNF network’.

IL-6 is another key regulator of the cytokine network in ovarian cancer cells and treatment of ovarian cancer patients with an anti-IL6 therapeutic antibody has also been shown to have some clinical activity, with periods of disease stabilization in some patients, reduced systemic cytokine and c-reactive protein levels. However these effects were short-lived patients ultimately progressed [9].

The molecular pathways stimulated in malignant cells through tumor-promoting cytokines activate transcription factors such as NFκB, STAT3, and HIF1α. These, in turn, control the production of other chemokines and inflammatory mediators [6]. Further studies are required to determine the critical signaling nodes or pathways in this robust inflammatory cytokine network that help maintain the oncogenic phenotype.

Here we have used a systems biology approach, integrating genomic and proteomic analyses, to determine a hierarchy of critical mediators and pathways associated with the TNF network that could be targeted in cancer. We identified a number of kinases associated with this inflammatory cytokine network and have validated one of these kinases, casein kinase 2 (CK2), as a potential driver of this network.

## RESULTS

### Identification of the kinase signaling cascades associated with the TNF network

**Figure 1:**
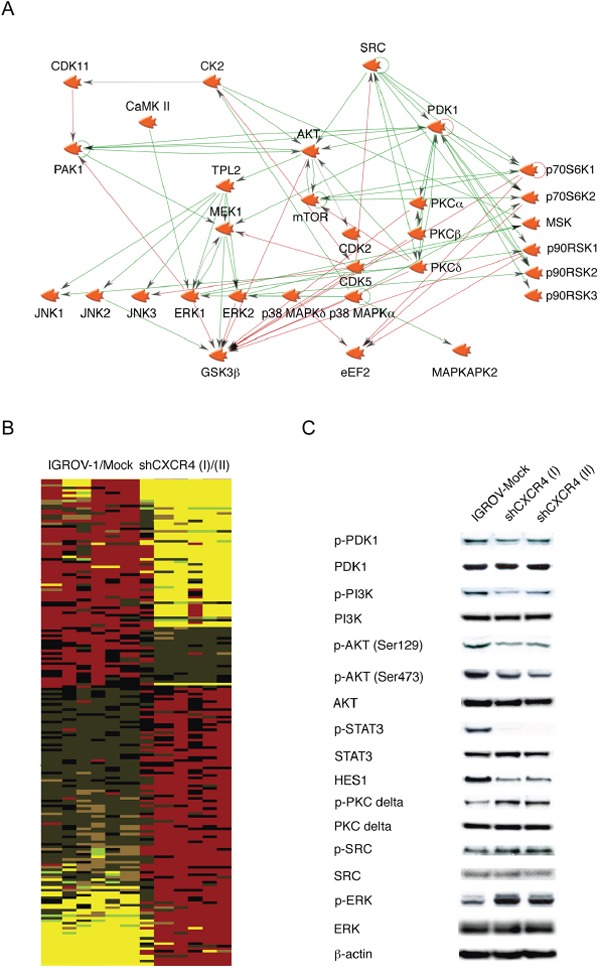
Phosphoprotein profiling of human ovarian cancer cell lines **A.** A network of putative constitutively active kinases in IGROV-1 cells was created using Genego's Metacore pathway analysis tool. Interactions between any two objects are depicted by an arrow indicating the direction of interaction. **B.** Heatmap containing three samples in each group of statistically significantly differentially phosphorylated proteins were generated using IGROV-1 and IGROV-Mock transfected versus two independently isolated clones of IGROV-1 cells stably transfected with shRNA to CXCR4 (I and II). Increases in phosphorylation are shown as pseudocolour red and decreases in yellow. **C.** Western blot analysis of selected kinases associated with the phenotype of high cytokine expression and downstream effect on phospho-STAT3 and NOTCH signaling in malignant cells.

To determine critical mediators in the signaling pathways associated with the TNF network, we established phosphoproteomic profiles using mass spectrometry analysis (LC-MS/MS) in a high TNF network expressing ovarian cancer cell line IGROV-1. We analyzed the constitutively active kinases in IGROV-1 cells through their respective substrates, using Kinase-Substrate Enrichment Analysis (KSEA) [25]. Of 45 constitutively active kinases, 33 of these showed direct interactions with each other according to MetaCore's Genego pathway analysis tool [GeneGo, Inc, St. Joseph, MI] (Figure 1A). Many of these kinases are involved in the deregulated activation of the MEK/ERK and PI3K/AKT pathway, including PDK1 and Casein kinase II, CK2, with involvement in chemotherapeutic drug resistance, proliferation of cancer initiating cells and angiogenesis [27]. We hypothesized that if there are significant differences in the activation of specific kinases associated with the TNF network, we should detect an increased phosphorylation of their respective substrates.

In the past we have shown that the chemokine receptor CXCR4 expressed by the malignant cells sustains the TNF network through autocrine interaction with its ligand CXCL12, as knockdown of this chemokine receptor drastically decreased TNF, IL6 and VEGF levels in cell culture supernatant of these cells [8]. Therefore we generated phosphopeptide signatures by comparison of phosphopeptide sites in IGROV-1 cells with cells in which the TNF network had been inhibited by shRNA to CXCR4. The phosphopeptide intensities obtained from the MS data were normalized to the median and log2 transformed. Phosphopeptides were considered significantly different across cell lines at the p<0.05 level by a t-test of log transformed data after Benjamini-Hochberg correction for multiple hypothesis testing [28]. The results are shown as a heatmap (Figure 1B), with yellow indicating lower and red indicating higher phosphorylation. Identified phosphorylation sites were linked to kinases acting upstream using Kinase-Substrate Enrichment Analysis [25].

An examination of the putative upstream kinases showed that a number of these were activated in association with the TNF network. Among these, PDK1 and CK2 showed increased activity in high cytokine producing IGROV-1 cells compared with its TNF network inhibited form (shCXCR4 cells). Since PDK1 and CK2 are involved in the regulation of the PI3K/AKT pathway, we have confirmed these findings by visualizing the phosphorylation of PDK1 and the CK2 specific phosphorylation of AKT (Ser129), a phosphorylation site that is often used as a marker of CK2 activity by Western blot analysis (Figure 1C) [29, 30]. Phosphorylation of PDK1 (Ser241), PI3K (Tyr458), AKT (Ser473 and Ser129), were reduced in the shCXCR4 cells in which the TNF network has been inhibited. We postulated that these associated kinases might be critical members of the TNF signalling network and therefore potential therapeutic targets.

Conversely, LC-MS/MS analysis showed that phosphorylation of PKC delta and its downstream target MARCKS were increased in shCXCR4 cells compared with IGROV-1 and mock-transfected control cells by LC-MS/MS analysis. We also detected an increase in phospho-ERK and the substrates for SRC by LC-MS/MS. However, the increased phosphorylation of SRC, when visualized by Western blot analysis was subtle (Figure 1C). This might be due to the greater sensitivity when LC-MS/MS was used for detection.

We previously identified key inflammatory pathways and NOTCH signalling in cancer promoting processes by Gene Set Enrichment Analysis (GSEA) using the high TNF network gene expression signature [2]. Here, we performed additionally GSEA using the phosphoprotein signature identified in these cells (Figure 1B) for process and pathways enrichment. Predominant in both analyses, with phosphoproteomics and gene expression data, NOTCH signalling was the most significantly enriched pathway (p<0.0001 and p=0.0003, respectively) in association with the TNF network. Furthermore, we have shown that NOTCH3 and its ligand, Jagged1, were regulated in a STAT3 dependent manner [2]. Again, the phosphorylation of STAT3 and the transcription factor HES1, downstream of NOTCH signalling, were also decreased in shCXCR4 cells compared to the mock-transfected control (Figure 1C).

Having identified the same important processes and pathways with both the proteomic and gene expression analyses, we next investigated the key kinases involved in regulation of the signaling pathways by exploring the gene expression signature of the TNF network further.

### Identification of therapeutic targets using a gene expression signature of the inflammatory phenotype

The interaction of a drug or chemical with a biological system can result in a characteristic gene-expression profile or signature. These signatures can be used to connect molecules with similar pharmacological or toxicological properties by gene expression signature matching. Lamb *et al* first proposed the Connectivity Map (cMap) to make successful connections among small molecules, genes, and diseases using genomic signatures [31]. Further studies showed that the cMap can be exploited to investigate the mode of action (MoA) of novel compounds and to identify new potential use for already approved drugs (i.e. drug repositioning) [32-34].

**Figure 2:**
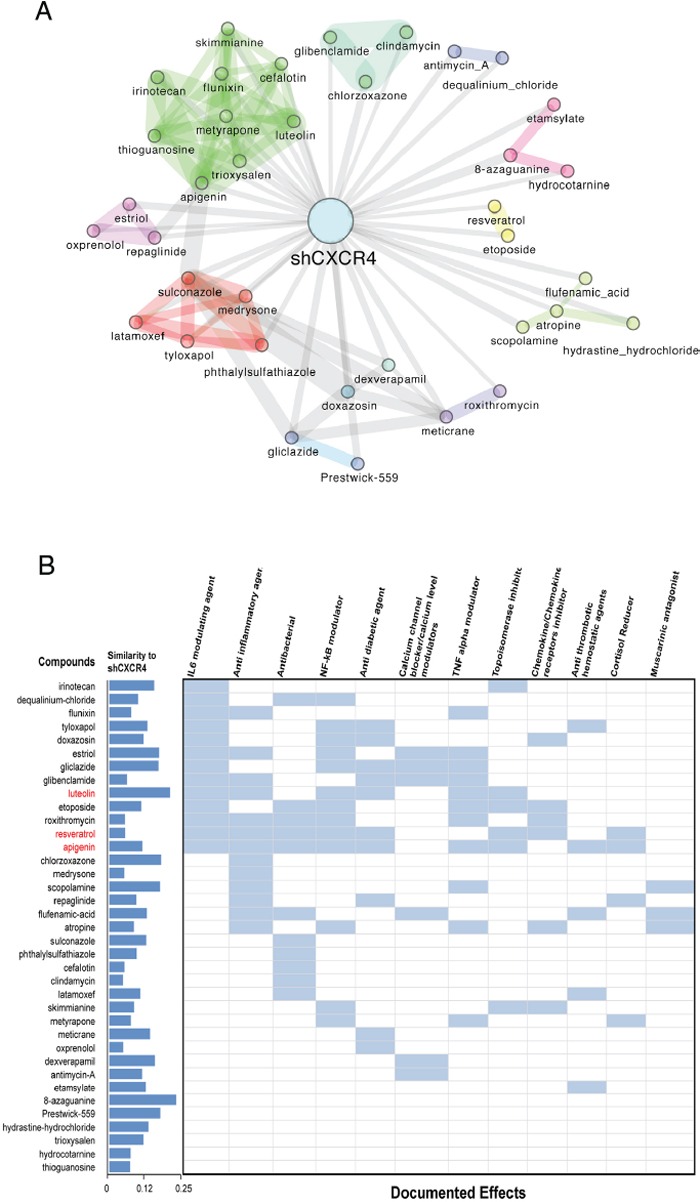
Summary of compounds whose transcriptional response is similar to that induced by the knock-down of the TNF Network **A.** Drug-network based classification using the gene expression signature in shCXCR4 cells. The expression signature of cells in which the TNF network has been inhibited is connected to a given drug if the profile of differential expression following the knock-down is significantly similar to the transcriptional response elicited by that drug in human cancer cell lines. Two drugs are connected to each other if their transcriptional response is similar according to the same criteria. Colors indicate groups of densely interconnected nodes enriched for a given mode of action, common drug targets or therapeutic application. **B.** Included are documented effects for each drug in light blue and shown in red are the drugs known to target protein kinase CK2. The dark blue bars indicate the extend of similarity between the transcriptional responses elicited by the listed compounds and that of the knock-down of CXCR4 (shCXCR4). This is defined as the inverse of the distance reported in Supplementary Table 1, and normalised in [0,1]. According to this metric the compound eliciting the most similar response to shCXCR4 is 8-azaguanine and CK2 is a recurrent target among those of the top-similar to shCXCR4.

To further investigate how to target the TNF network, we mapped the gene expression data, using the gene expression signatures of CXCR4 knockdown cells versus mock-transfected cells, onto the Connectivity Map of drugs, in order to identify any compounds having an effect on transcription similar to that of the knock-down of the TNF network. We made use of the Mode of Action by Network Analysis (MANTRA) computational framework proposed in [32]. Through this tool we integrated the profile of differential expression following the CXCR4 knock-down into a drug similarity network containing all the cMap compounds. This analysis led to a network of compounds ‘connected’ to the CXCR4 knock-down profile of differentially expressed genes (Figure 2A and Supplementary Table 1A-F). According to the MANTRA tool, these compounds elicit a transcriptional response similar to that triggered by inhibiting the TNF network by knocking-down CXCR4 and are clustered in ‘network communities’, statistically enriched for certain MoAs.

A summary of these compounds and some of their known effects is shown in Figure 2B. Many of these compounds have immune modulatory properties. Among the identified drugs were luteolin, apigenin (members of the same drug community) and resveratrol. One of the known targets of this class of drugs is CK2, a protein kinase that we identified by phosphoproteomics and KSEA to be associated with the TNF network (Figure 1A and 1C) [35]. Furthermore, resveratrol has been reported to suppress TNF induced activation of NFκB and AP-1, and to suppress proliferation, as well as being able to overcome chemoresistance through the down-regulation of STAT3 [36-38].

We therefore hypothesised that CK2 was a key driver of the TNF network and hence specific inhibition of this kinase might inhibit the activation of the PI3K/AKT pathway and the transcription of downstream targets, such as TNF and IL6.

### Validation of CK2 as a potential target

**Figure 3:**
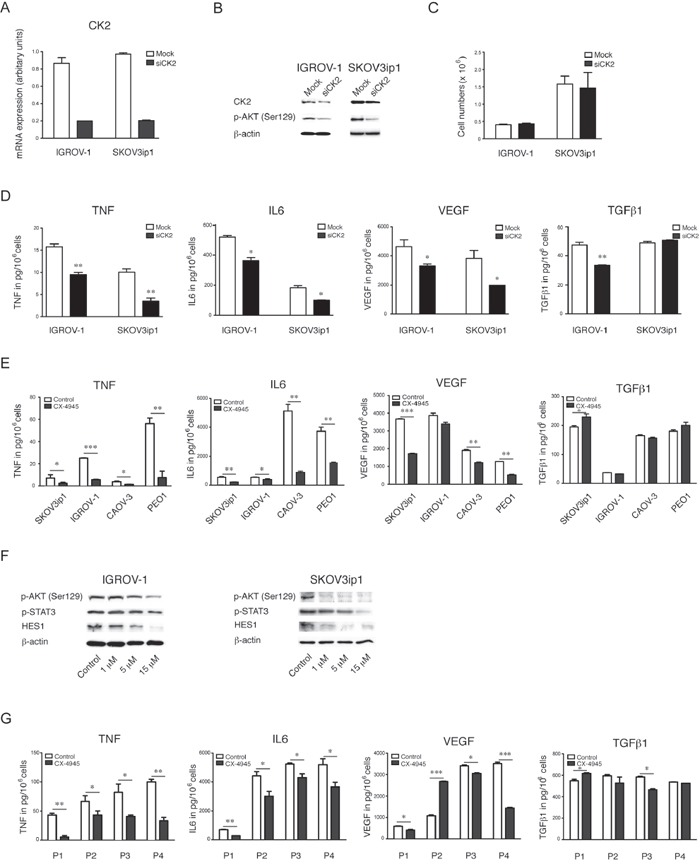
Effects of specific inhibition of CK2 on the cytokine network in EOC **A.** mRNA gene expression of CK2 by real time RT-PCR, **B.** Western blot analysis **C.** proliferation and **D.** cytokine production after 48 hours of transient transfection with a pool of 3 oligos targeting CK2 in IGROV-1 and SKOV3ip1 cells (mean ± SEM, **, P<0.01, *, P<0.05). **E.** Cytokine and growth factor expression levels were measured in cell culture supernatants after 48 hours inhibition of CK2 with 5 μM CX-4945. **F.** Western blot analysis of constitutive CK2 activity by its substrate phospho-Akt (Ser129) and downstream effects after treatment with CX-4945 in a dose dependent manner after 24hour treatment with and without CX-4945 (mean ± SEM, ***, P<0.001, **, P<0.01, *, P<0.05). **G.** Cytokine and growth factor expression levels in cell culture supernatants of primary ovarian cancer cells from ascites after 48 hours inhibition of CK2 with 5 μM CX-4945.

In order to validate CK2 as a potential driver or mediator of the TNF network, we directly inhibited the kinase activity by silencing CK2 expression, using siRNA, in two ovarian cancer cell lines, IGROV-1 and SKOV3ip1, with similar constitutive TNF, IL6 and VEGF secretion into cell culture supernatants. As shown in Figure 3A, almost 80% knockdown of CK2 mRNA expression was achieved after a 48hour transfection with a pool of specific siRNA oligonucleotides. The decrease of CK2 activity in both cell lines after knockdown with siRNA oligos was monitored via its known substrate of AKT (Ser129) by Western blot, as shown in Figure 3B. TNF, IL6 and VEGF cytokine production was significantly inhibited after transfection (Figure 3D) with no effect on cell proliferation (Figure 3C).

In order to confirm the findings observed in our CK2 knockdown experiments we next determined the effects of the specific CK2 inhibitor CX-4945, which is currently under investigation in clinical trials [39, 40]. Treatment with CX-4945 significantly reduced the constitutive release of TNF and IL6 was significantly reduced in the cell culture supernatant of five ovarian cancer cell lines that were cytokine producers after treatment with CX-4945 (Figure 3E) and VEGF production was also inhibited in 3/4 parental cell lines (Figure 3E). CX-4945 inhibited the JAK/STAT3 pathway and protein levels of the NOTCH transcription factor HES1 in both IGROV-1 and SKOV3ip1 cells (Figure 3F). Finally, we observed a significant decrease in TNF and IL6 production following CK2 inhibition with CX-4945 by ELISA in cell culture supernatant of primary cells derived from ascites of four high-grade serous ovarian cancer patients (Figure 3G). Levels of VEGF were also inhibited in three out of four of these primary cancer cell supernatants after treatment with CX-4945 (Figure 3G).

As TGFβ1 was not associated with the TNF network [2] we included this as an internal negative control. Levels of this cytokine did not change significantly as a function of CK2 activity (Figure 3D, 3E and 3G).

### Apoptotic and anti-proliferative activity of CK2 inhibition by CX-4945 *in vitro*

**Figure 4:**
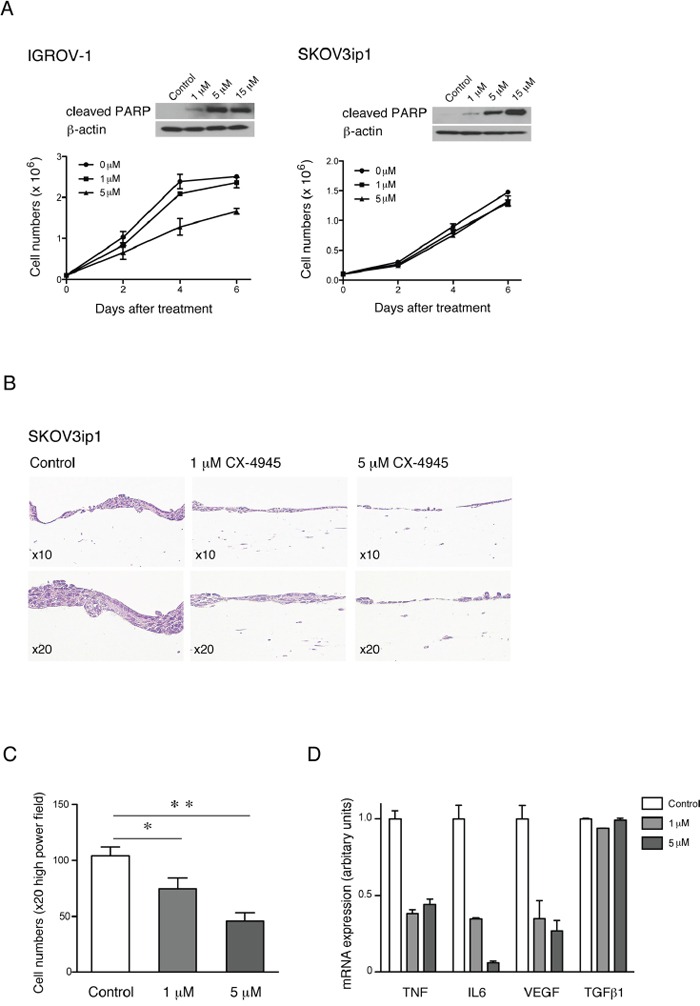
Effects on apoptosis and proliferation of HGSC cell lines after specific inhibition of CK2 *in vitro* Apoptotic and proliferative effect of CK2 inhibitor CX-4945 on **A.** IGROV-1 and SKOV3ip1 cells, in a dose dependent manner by Western blot analysis of apoptotic marker cleaved PARP and by cell numbers on plastic over a period of 6 days, and **B.** representative pictures of H&E stained sections in a 3D model after 6 days treatment, respectively. **C.** Quantification of cell growth inhibition and **D.** mRNA expression of cytokines and growth factors after treatment of SKOV3ip1 cells with CX-4945 in the 3D model (mean ± SEM, **, P<0.01, *, P<0.05).

In order to determine the apoptotic activity of CX-4945 in IGROV-1 and SKOV3ip1 cells, we performed Western blot analysis of cleaved PARP. The results demonstrate an increase in apoptosis in both ovarian cancer cell lines after inhibition of CK2 with CX-4945 in a dose dependent manner (Figure 4A).

To assess the effect of CK2 inhibition and anti-proliferative activity of CX-4945 we performed proliferation assays on plastic and in a 3-dimensional (3D), organotypic model of ovarian cancer. Treatment of IGROV-1 cells with 5μM CX-4945 over a period of 6 days showed a modest reduction in proliferation (28%) but had no effect on proliferation of SKOV3ip1 cells (Figure 4A) on plastic despite the effect seen on apoptosis by cleaved PARP by Western blot analysis. These results are consistent with previous studies, in which the TNF network was inhibited by shRNA to CXCR4 or TNF in ovarian cancer cells [2, 8]. However, the proliferation of SKOV3ip1 cells were significantly reduced in a dose dependent manner after treatment with 1 μM and 5 μM CX-4945 over the same period of days in a 3D organotypic model assay (Figure 4B and 4C). Moreover mRNA expression levels of key members of the TNF network were also significantly reduced in this model. Again, TGFβ1 was included as an internal control and mRNA expression levels of this cytokine did not change in this setting (Figure 4D).

### Specific inhibition of CK2 *in vivo*

**Figure 5:**
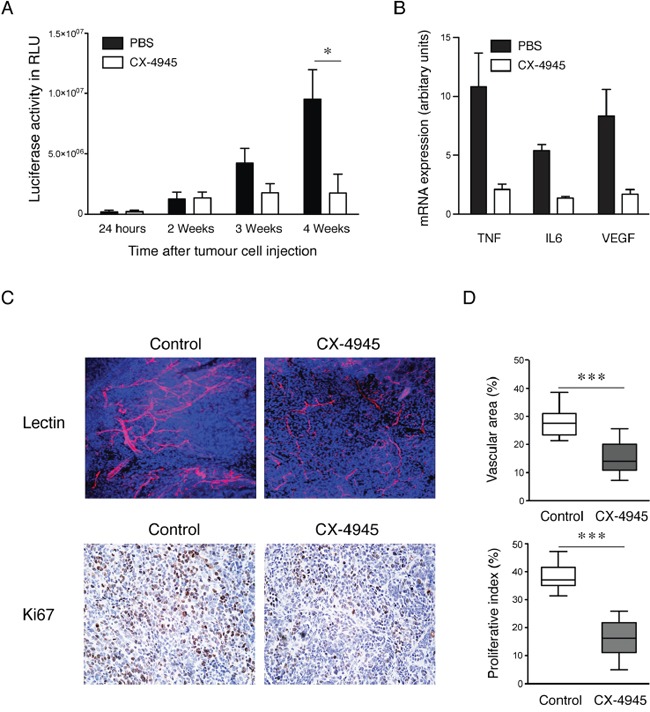
Effects of specific inhibition of CK2 on xenograft growth *in vivo* **A.** Quantification of bioluminescence from tumors (n=6 mice per group) at different time points (*, P <0.05) and **B.** mRNA expression of cytokines in primary tumors (n=3) by real time RT-PCR after treatment with CK2 inhibitor CX-4945. **C.** Representative pictures of confocal images (magnification x20) from tumors following injection of TRITC-lectin and Ki67 stained paraffin sections by immunohistochemistry after 42 days treatment with 75mg/kg CX4945. **D.** Quantification vascular area and proliferative index. Columns, mean in each group in 10 randomly selected areas of tumor sections (mean ± SEM, ***, P<0.001).

Treatment with CX-4945 (75mg/kg) resulted in a significant reduction in tumor growth of the peritoneal ovarian cancer xenograft IGROV-1 after 42 days as measured by bioluminescence imaging (Figure 5A). The cytokine mRNA expression levels of TNF, IL6 and VEGF were significantly lower in tumors from CX-4945 treated animals (Figure 5B). This correlated with significantly decreased vascular area of size-matched tumor deposits compared with the control group and reduced proliferative index of the malignant cells as assessed by immunohistochemistry for Ki67 (p<0.001) (Figure 5C and 5D).

## DISCUSSION

Malignant cells from many different cancer types constitutively produce inflammatory cytokines and chemokines that are important in tumor growth and cancer progression, TNF and IL-6 being the most commonly reported [41, 42]. The mediators are co-regulated in complex networks and are downstream of many common oncogenic mutations, but key nodes in intracellular signaling networks that control this constitutive production are not clearly defined [3, 5]. The approach described here has allowed us to identify casein kinase II, CK2, as a regulator and potential intracellular target to inhibit the inflammatory phenotype in cancer.

To conduct this study we used cancer cell lines that produced high levels of TNF network proteins. It is now clear that the term ‘ovarian’ cancer refers to at least five distinct diseases all of which grow and spread within the peritoneal cavity and ovary [43]. These five cancers; low-grade serous, mucinous, endometrioid, clear cell and high-grade serous, are now defined by their genomic profile and biomarkers. This concept has major implications for human cell line models of ovarian cancer as recent analysis of commonly used ovarian cancer cell lines, including some of those used in this paper, has questioned which type of ovarian cancer they may represent [14, 15]. Some of the cell lines we have used, as well as the primary malignant cells, are derived from the most lethal and common form of ovarian cancer, high-grade serous. Clear cell carcinoma cells also produce high levels of IL-6 [15] and other TNF network members (our unpublished data). Therefore we believe that our analysis of the regulation of the TNF network by CK2 is relevant to several different epithelial cancer types and may also be relevant to other malignancies such as myeloma [44].

CK2 is a highly conserved constitutive active serine/threonine protein kinase that regulates multiple pathways frequently activated in cancer including the PI3K/AKT, RB, and WNT signaling cascade [45, 46]. Through phosphorylation of more than 300 substrates, it is involved in DNA replication, gene transcription, signal transduction, cell growth and apoptosis [47, 48]. Although CK2 is overexpressed in myeloma [49], leukemia [50] and solid tumors including lung, kidney and head and neck cancers [46] it is not *per se* a classical oncogene. However, CK2 was shown to be essential in maintaining the phenotype of inflammatory breast cancers [39, 40] and has been recently described as therapeutic target in PTEN-deficient tumors [30]. Even though PTEN is only mutated in about 7% of HGS ovarian cancers, PTEN deletion or downregulation is much more frequent than to be thought and therefore its impact on the PI3K/AKT pathway might be crucial in up to 50% of HGS ovarian cancers [51].

We validated CK2 as a potential driver of the inflammatory phenotype in ovarian cancer cells using siRNA and the selective small molecule inhibitor CX-4945 of CK2 expression levels and activity respectively. Knockdown of CK2 expression in malignant cells by siRNA or treatment with the specific CK2 inhibitor CX-4945 reduced significantly cytokine expression *in vitro* and decreased Notch signaling. Even though we did not see an anti-proliferative effect by inhibition of the kinase with neither siRNA oligos nor specific inhibitor CX-4945 on plastic, there was a significant reduction of cell numbers in a 3D organotypic model of ovarian cancer with CX-4945. The expression level of TNF, IL6 and VEGF was also inhibited after treatment with CX-4945 in this model. Therefore it is possible that these cytokines are inducing other cytokines and growth factors in the fibroblast and mesothelial cells to stimulate tumor growth. Hence, inhibition of CK2 by CX-4945 might lead to the decrease in cell numbers by blocking the paracrine action of the cytokines on stromal cells in 3D cultures. Proliferative activity of the malignant cells was also inhibited by CX-4945 *in vivo* therefore this might be a key mechanism in peritoneal ovarian tumor growth.

Surprisingly, we also noticed an increase in activity of PKC and the tyrosine kinase SRC after inhibition of the TNF network (Figure 1C). In general, kinase inhibitors have had limited success in cancer treatment as single agent therapies, despite the biological effects achieved in preclinical studies. The reasons may be that cancer cells present an enormous genetic heterogeneity and circumvent their action through a robust signaling network with a reciprocal regulatory feedback mechanism. Therefore, PKC and/or SRC are maybe promising therapeutic targets in combination with CK2 inhibition in ovarian cancer and we will investigate this in future studies.

In conclusion, we have identified kinases, particularly CK2, associated with the TNF network that may play a central role in sustaining the cytokine network and/or mediating its effects in ovarian cancer.

## MATERIALS AND METHODS

### Ovarian cancer cells

Ovarian cancer cell lines SKOVip1, IGROV-1, CAOV-3, and PEO1 were selected for high expression levels of key members of the TNF network and cultured as described [8]. All cell lines have undergone 16 loci STR authentication (LGC Standards, London, UK). The cells were cultured in RPMI 1640 10% FCS, routinely tested for mycoplasma contamination and only passaged four times before new stocks were recovered from liquid nitrogen stores. Recent studies identified CAOV3 and PEO1 as HGSC cell lines. However, the widely used cell lines SKOV-3 and IGROV-1 were implicated as not being representative of the major HGSC subtype [14-16]. All primary cells were isolated from human ascites collected from ovarian cancer patients at St. Bartholomew's hospital. All samples were taken under the guidelines of the Human Tissue Authority Act 2004 and only from patients whom had given prior consent under the Research Ethical Committee Project reference 10/H0304/14.

### Transfection of IGROV-1 and SKOV3ip1 cells

Cells were transfected with the ON-TARGET plus SMART pool of oligos targeting CSNK2A1 gene expression using Lipofectamin as described previously [8]. SiCONTROL non-targeting siRNA pool served as control.

### Western blotting

Cell extract (15μg) was run on an SDS 10% acrylamide gel and transferred to a nylon membrane. The membrane was blocked for 1hour (4°C in PBS with 0.1% Tween and 10% milk powder) and probed overnight using anti-phospho-PDK1 (3061, Cell Signaling, UK), PDK1 (3062, Cell Signaling, UK), phospho-PI3K (4228, Cell Signaling, UK), PI3K (4255, Cell Signaling, UK), phospho-Akt473 (sc-9271, Santa Cruz, UK), Akt473 (4685, Cell Signaling, UK), phoshor-STAT3 (D3A7, Cell Signaling, UK), STAT3 (4904, Cell Signaling, UK), anti-Casein kinase 2 (06-873, Millipore, UK), phospho-Akt129 (AP3020, Cambridge Biosience, UK), and HES1 (H140, Santa Cruz, UK). A horseradish peroxidase-conjugated secondary antibody was used for detection (1:2,000) dilution at room temperature for 1 hour. Protein concentration equivalence was confirmed by anti-β-actin antibody.

### Cytokine ELISA

Cells were plated at 3 × 10^5^ cells/well, cell culture supernatants removed after 48 hours of culture and cytokine concentrations measured using Quantikine^®^ ELISA kits (R&D Systems) or Cytokine analyses with ECL assays were done according to man-manufacturer's instructions (MSD human IL6, TNF, IL8, and VEGF multiplex microplate, N45CA-1).

### RNA extraction and real time quantitative RT-PCR

RNA was extracted using Tri Reagent (Sigma), and treated with 10 U DNase (Pharmacia, Milton Keynes, UK). DNase treated RNA (2 μg) was reverse transcribed with M-MLV reverse transcriptase (Promega, Southampton, UK). Multiplex real-time RT-PCR analysis was performed using pre-made TaqMan^®^ probes (FAM) and 18s rRNA (VIC) specific primers and probes with the ABI PRISM 7700 Sequence Detection System instrument and software (PE Applied Biosystems, Warrington, UK). Expression values were normalized (ΔCt) to 18s rRNA by subtracting the cycle threshold (Ct) value of 18s rRNA from the Ct value of the experimental value.

### Phosphoproteomics

Mass spectrometry based phosphoproteomics was performed as described before with minor modifications [17, 18]. Briefly, cells were lysed in urea lysis buffer (8M urea in 20 mM HEPES pH 8.0) containing phosphatase inhibitors (1 mM Na_3_VO_4_, 1 mM NaF, 1 mM β-glycerol phosphate, 1.25 mM sodium pyrophosphate). After centrifugation (20,000g for 5 min at 5°C), protein levels quantified by Bradford analysis. A sample aliquot containing 500 μg of protein was diluted to a final volume of 1 ml in denaturing buffer and 4.1 mM dithiothreitol and 8.3 mM iodoacetamide sequentially added for 15 min at room temperature in the dark. After dilution of samples to a final concentration of 2 M urea using 20 mM HEPES (pH 8.0), proteins were digested with TPCK-Trypsin (20 TAME units/mg) for 16 h at 37°C. The resultant peptide solutions desalted by solid-phase extraction with Oasis reversed phase solid phase extractions columns (Waters UK ltd, Manchester, UK) following the manufacturer's instructions with modifications. Elution solution contained 60% acetonitrile (ACN), 2M glycolic acid, 5% TFA.

### Phosphopeptide extraction and analysis

Phosphopeptides were enriched by TiO_2_ chromatography as described by Larsen [19] with the modifications described elsewhere [17] and analysed in a LC-MS/MS system in random order to remove potential batch effects. Phosphoprotein separation was performed in a nanoflow ultra-high pressure liquid chromatography system (nanoAcquity, Waters) using a BEH 100 μm x 100 mm column (Waters) and a binary mobile phase gradient with 0.1% formic acid in LC-MS grade water (A) 0.1% formic acid in LC-MS grade acetonitrile (solution B). Gradients used were as follows: 1% B for 5 min, 1% B to 35% B over 100 min, a 5 min wash at 85% B and a 7 min equilibration step at 1% B. Gradient elution was at 400 nl/min. An LTQ-Orbitrap XL mass spectrometer (Thermo Fisher Scientific, Hemel Hempstead, UK) acquired full scan survey spectra (m/z 350–1600) with a resolution of 30,000 at m/z 400 and the top five most abundant multiply charged ions present in each survey spectrum were fragmented by collision-induced dissociation (CID, normalized collision energy 35%) with multi stage activation (MSA). A dynamic exclusion of 40 sec and mass window of 10 ppm was used.

### Identification and quantitation of phosphopeptides

Identification and quantification of phosphopeptides from LC-MS/MS data was as described before [20, 21]. Briefly, Mascot Distiller 2.3.2 was used to smoothen and centroid the MS/MS data and to generate peak list files which were searched against the human sequence library in the SwissProt database (version 2010_03 containing 23,000 entries, http://expasy.org/sprot/) using the Mascot search engine [22]. Search parameters included: trypsin as digestion enzyme with two missed cleavages allowed, carbamidomethyl (C) as fixed modification, and Pyro-glu (N-term), Oxidation (M) and Phospho (STY) as variable modifications. A mass tolerance of ± 7 ppm for the precursor ion and ±800 mmu for fragment ions was allowed. Hits were considered significant when they had an Expectation value of < 0.05 (as returned by Mascot). False discovery rates were ∼0.02 as determined by decoy database searches. Sites of modification were reported when the Mascot delta score was greater than 10 [23]; otherwise site assignment was deemed ambiguous and are reported as gene name followed by phosphorylation site within the protein sequence and charge of the measured ion. Phosphopeptides identified by Mascot above the statistically significant threshold were selected for quantification with Pescal [18, 24]. The resulting quantitative data were parsed into Excel files for further normalization and statistical analysis. Phosphopeptide intensities were normalized to the total chromatogram intensity and to the mean value across samples. In order to infer kinase activity from quantitative phosphoproteomics data, phosphorylation sites were linked to kinases acting upstream using Kinase-Substrate Enrichment Analysis [25].

### 3-dimensional (3D) organotypic model

The method was used as previously described [26] with minor modifications. All primary cells were isolated from human omentum samples collected from surgery on ovarian cancer patients at St. Bartholomew's hospital. Samples were taken under the guidelines of the Human Tissue Authority Act 2004 and only from patients whom had given prior consent under the Research Ethical Committee Project reference 10/H0304/14. For the 3D Omental model construction, a collagen mix was made up on ice constituting one part 10X RPMI-1640 (powder dissolved in distilled water and Sodium Bicarbonate; Sigma, R6504), one part 0.33M Sodium Hydroxide, (Sigma, S-8045), one part FBS, and seven parts Rat-tail Collagen, Type 1, (BD Biosciences, 354236). 50μL of the mix was added to each well of a 24 well Corning Transwell-Clear, plate 12 inserts, 3μm pore size, 6.5mm diameter, (Fisher, TKT-525-090R), and allowed to set for 30 minutes. 50,000 human embryo fibroblasts (HEF) were mixed into 50μL of the collagen mix per well, and allowed to set on top of the pre-set layer. 500μL and 100μL of full medium were placed in the bottom and top part of the transwell respectively and left in culture for 24 hours. 40,000 human primary mesothelial cells (HPMC) were then seeded in 100μL of full medium to the top part of each transwell and left to culture for 24 hours. 50,000 ovarian cancer cells were then seeded in 100μL of full medium to the top part of each transwell and left to culture for a further 24 hours. The top and bottom compartment of each transwell was washed in PBS, and full medium was placed in the bottom compartment and serum-free medium was placed in the top compartment to generate a serum concentration gradient. The models were left in culture for 14 days with and without CK2 specific inhibitor CX4945 (Cylene).

### Growth of human ovarian cancer cell lines *in vivo*

BALB/C nude female mice (Charles River, UK) 6-8 weeks of age, were used in all experiments. Mice were housed in sterile individually ventilated cages (IVC) at 20°C. Mice were injected i.p. with 1 × 10^7^ IGROV-1/Luciferase cells, observed daily for tumor growth and were killed when peritoneal swelling reached the endpoint stated in our Home Office licence PPL 70/6578 (20% increase in abdominal girth). Paraffin embedded sections were stained with an anti-Ki67 antibody (SC-7846, Santa Cruz Biotech).

### Bioluminescence imaging

Mice were injected i.p. with 150 μg/g bodyweight D-luciferin in PBS, and bioluminescence imaging with a charge-coupled device (CCD) camera (IVIS, Xenogen, Alameda, CA) was initiated 10 minutes after injection (Smith et al. 2004). Bioluminescence images were obtained with a 15 cm field of view (FOV), binning (resolution) factor of 8, 1/f stop, and open filter with an imaging time of 5 seconds. Data were analyzed using Living Image software (also Xenogen) and are presented as relative light units (RLU) of light emission/s/cm^2^ from ventral imaging and photon flux from a ROI drawn over a mouse that was not given an injection of luciferin.

### Quantification of tumor blood vessels

To visualize the architecture of blood vessels, animals were anesthetised with isoflurane, injected with TRITC-conjugated Lycopersicon Esculentum (tomato lectin) (100 μl, 2 mg/ml, Vector Laboratories, Burlingame, CA) via the tail vein 3 min before animals were perfused with 4% paraformaldehyde. Following fixation overnight in 4% PFA resected primary tumors were cryoprotected in 12%, 15%, and 18% sucrose for 1hour each. Tumors were subsequently snap-frozen in O.C.T. compound (Sankura Finetek, Torrance, CA) and sectioned at 50 μm intervals. Vessels were visualized using confocal microscopy (Zeiss LSM S10 META) and microvessel density quantified with ImagePro Plus software (Image-Pro plus, Media Cybernetics, Silver Spring, MD). Microvessel density was expressed as mean percentage of microvessel surface area.

### Statistical analysis

Statistical analysis of *in vitro* and animal experiments used one-way ANOVA or unpaired T-test with Welch correction (GraphPad Prism version 4 Software, San Diego, CA).

## SUPPLEMENTARY TABLES




