# Supplementary material for: Integrated transcriptomic and proteomic analysis identifies protein kinase CK2 as a key signaling node in an inflammatory cytokine network in ovarian cancer cells
Source: Oncotarget. 2016 Feb 8;7(13):15648–61. doi: 10.18632/oncotarget.7255 (PMC4941267; doi:10.18632/oncotarget.7255)
Supplement: Supplementary file 1 [file oncotarget-07-15648-s001.pdf]

SUPPLEMENTARY TABLES

Supplementary Table S1A: List of compounds whose transcriptional response is similar to that induced by the knock-down of the TNF Network

See Supplementary File 1

Supplementary Table S1B: Annotations and documented effects for the list of compounds whose transcriptional response is similar to that induced by the knock-down of the TNF Network

See Supplementary File 1

Supplementary Table S1C: Enriched drug features for drug community n. 32 in the MANTRA neighbourhood of shCRXCR4

See Supplementary File 1

Supplementary Table S1D: Enriched drug features for drug community n. 13 in the MANTRA neighbourhood of shCRXCR4

See Supplementary File 1

Supplementary Table S1E: Enriched drug features for drug community n. 42 in the MANTRA neighbourhood of shCRXCR4

See Supplementary File 1

Supplementary Table S1F: Enriched drug features for drug community n. 89 in the MANTRA neighbourhood of shCRXCR4

See Supplementary File 1
