# Supplementary material for: Integrated transcriptomic and proteomic analysis identifies protein kinase CK2 as a key signaling node in an inflammatory cytokine network in ovarian cancer cells
Source: Oncotarget. 2016 Feb 8;7(13):15648–61. doi: 10.18632/oncotarget.7255 (PMC4941267; doi:10.18632/oncotarget.7255)
Supplement: Supplementary file 2 [file oncotarget-07-15648-s002.docx]

Supplementary Table 1A

| **Compound** | **Distance from**  **shCRXCR4** | **ATC-Codes** | **Direct Target** | **Biochemical Interactions** |
| --- | --- | --- | --- | --- |
| 8-azaguanine | 0.7313 |  |  |  |
| luteolin | 0.7396 |  | CSNK2A1 | acts as an inhibitor of protein topoisomerase (DNA) I |
| chlorzoxazone | 0.7509 | M03BB03 | KCNMA1 |  |
| Prestwick-559 | 0.7521 |  |  |  |
| scopolamine | 0.7524 | A04AD01, N05CM05, S01FA02 | CHRM1 |  |
| estriol | 0.7537 | G03CA04, G03CC06 | ESR1 cyp51 |  |
| gliclazide | 0.7546 | A10BB09 | KCNJ1 ABCC8 ALB VEGF |  |
| dexverapamil | 0.7594 |  |  | acts as an inhibitor of protein Voltage-dependent L-type calcium channel acts as an inhibitor of protein Voltage-dependent L-type calcium channel acts as an inhibitor of proteins with low voltage-gated calcium channel activity acts as a blocker of protein calcium channel, voltage-dependent, alpha 1G subunit |
| irinotecan | 0.7603 | L01XX19 | TOP1MT TOP1 | acts as an inhibitor of protein topoisomerase (DNA) Iacts as an inhibitor of protein topoisomerase (DNA) I |
| meticrane | 0.7659 | C03BA09 |  |  |
| Hydrastine  hydrochloride | 0.7682 |  |  |  |
| tyloxapol | 0.7696 | R05CA01 |  |  |
| Flufenamic acid | 0.7702 | M01AG03 | AKR1C3 TTR |  |
| sulconazole | 0.7711 | D01AC09 |  |  |
| etamsylate | 0.7716 | B02BX01 |  |  |
| doxazosin | 0.7748 | C02CA04 | ADRA1A ADRA1B ADRA1D | acts as an antagonist of protein adrenergic, alpha-1A-, receptor |
| trioxysalen | 0.7749 | D05AD01, D05BA01 |  |  |
| apigenin | 0.7763 |  | CSNK2A1 |  |
| antimycin_A | 0.7767 |  |  |  |
| etoposide | 0.7778 | L01CB01 | TOP2A MAP2K7 | acts as an inhibitor of protein DNA topoisomerase II |
| latamoxef | 0.7791 | J01DD06 |  |  |
| Dequalinium  chloride | 0.7822 |  |  |  |
| Phthalylsulfat-hiazole | 0.7840 |  |  |  |
| repaglinide | 0.7844 | A10BX02 | KCNJ1 ABCC8 KCNJ11 |  |
| skimmianine | 0.7869 |  |  |  |
| atropine | 0.7871 | A03BA01, S01FA01 | CHRM2 CHRM1 CHRM4 CHRM5 CHRM3 ACHE | acts as an antagonist of proteins with muscarinic acetylcholine receptor activity |
| flunixin | 0.7907 |  |  |  |
| metyrapone | 0.7912 | V04CD01 | camC CYP3A4 |  |
| hydrocotarnine | 0.7916 |  |  |  |
| thioguanosine | 0.7921 |  |  |  |
| glibenclamide | 0.7964 | A10BB01 | KCNJ1 ABCC3 ABCC9 ABCB11 ABCA1 CFTR ABCC1 ABCC8 KCNJ11 | acts as a blocker of proteins with ATP-activated inward rectifier potassium channel activity |
| resveratrol | 0.7989 |  | TTR NQO2 PTGS1 CSNK2A1 PTGS2 CHS2 | acts as an inhibitor of protein ribonucleotide reductase M1 polypeptide acts as an inhibitor of protein casein kinase 2, alpha 1 polypeptide acts as an inhibitor of proteins with prostaglandin-endoperoxide synthase activity |
| roxithromycin | 0.7992 | J01FA06 | rplJ ABCB1 |  |
| cefalotin | 0.7997 | J01DB03 | pbpA ALB CD248 ampC |  |
| medrysone | 0.8000 | S01BA08 | PLA2G4A SERPINA6 |  |
| oxprenolol | 0.8014 | C07AA02 | ADRB1 ADRB2 |  |
| clindamycin | 0.8017 | D10AF01, G01AA10, J01FF01 | rplJ |  |
| 8-azaguanine | 0.7313 |  |  |  |

**List of compounds whose transcriptional response is similar to that induced by the knock-down of the TNF Network.**

Supplementary Table 1B

| **Compound** | **Therapeutic Indications** | **Therapeutic Uses** | **Mode of Action** | **Pharmacology** |
| --- | --- | --- | --- | --- |
| 8-azaguanine |  |  |  |  |
| luteolin |  |  |  |  |
| chlorzoxazone |  | muscle relaxant (spasmolytic) | Chlorzoxazone inhibits degranulation of mast cells, subsequently preventing the release of histamine and slow-reacting substance of anaphylaxis (SRS-A), mediators of type I allergic reactions. Chlorzoxazone also may reduce the release of inflammatory leukotrienes. Chlorzoxazone may act by inhibiting calcium influx. | Chlorzoxazone, a synthetic compound, inhibits antigen-induced bronchospasms and, hence, is used to treat asthma and allergic rhinitis. Chlorzoxazone is used as an ophthalmic solution to treat conjunctivitis and is taken orally to treat systemic mastocytosis and ulcerative colitis. Chlorzoxazone is also a centrally-acting agent for painful musculoskeletal conditions. Data available from animal experiments as well as human study indicate that chlorzoxazone acts primarily at the level of the spinal cord and subcortical areas of the brain where it inhibits multisynaptic reflex a.c. involved in producing and maintaining skeletal muscle spasm of varied etiology. The clinical result is a reduction of the skeletal muscle spasm with relief of pain and increased mobility of the involved muscles. |
| Prestwick-559 |  |  |  |  |
| scopolamine |  | mydriatic | Scopolamine acts by interfering with the transmission of nerve impulses by acetylcholine in the parasympathetic nervous system (specifically the vomiting center). | Scopolamine is a muscarinic antagonist structurally similar to the neurotransmitter acetylcholine and acts by blocking the muscarinic acetylcholine receptors and is thus classified as an anticholinergic. Scopolamine has many uses including the prevention of motion sickness. It is not clear how Scopolamine prevents nausea and vomiting due to motion sickness. The vestibular part of the ear is very important for balance. When a person becomes disoriented due to motion, the vestibule sends a signal through nerves to the vomiting center in the brain, and vomiting occurs. Acetylcholine is a chemical that nerves use to transmit messages to each other. It is believe that Scopolamine prevents communication between the nerves of the vestibule and the vomiting center in the brain by blocking the action of acetylcholine. Scopolamine also may work directly on the vomiting center. Scopolamine must be taken before the onset of motion sickness to be effective. |
| estriol |  | hormonal agent | Estriol levels can be measured to give an indication of the general health of the fetus. DHEA-S is produced by the adrenal cortex of the fetus. This is converted to estriol by the placenta. If levels of "unconjugated estriol" are abnormally low in a pregnant woman, this may indicate a problem with the development of the child. | Estriol (also oestriol) is one of the three main estrogens produced by the human body. It is only produced in significant amounts during pregnancy as it is made by the placenta. In pregnant women with multiple sclerosis (MS), estriol reduces the disease's symptoms noticeably, according to researchers at UCLA's Geffen Medical School. |
| gliclazide |  |  | Gliclazide binds to the beta cell sulfonyl urea receptor (SUR1). This binding subsequently blocks the ATP sensitive potassium channels. The binding results in closure of the channels and leads to a resulting decrease in potassium efflux leads to depolarization of the beta cells. This opens voltage-dependent calcium channels in the beta cell resulting in calmodulin activation, which in turn leads to exocytosis of insulin containing secretorty granules. | Gliclazide is a second generation sulphonylurea which acts as a hypoglycemic agent. It stimulates beta cells of the islet of Langerhans in the pancreas to release insulin. It also enhances peripheral insulin sensitivity. Overall it potentiates insulin release and improves insulin dynamics. |
| dexverapamil | Tachycardia, VentricularHypertension Angina Pectoris | anti-arrhythmic anti-hypertensive vasodilator |  |  |
| irinotecan | Colorectal NeoplasmsNeoplasms | anti-neoplastic | Irinotecan inhibits the action of topoisomerase I. Irinotecan prevents religation of the DNA strand by binding to topoisomerase I-DNA complex, and causes double-strand DNA breakage and cell death. | Irinotecan is an antineoplastic enzyme inhibitor primarily used in the treatment of colorectal cancer. Irinotecan is a derivative of camptothecin. Camptothecins interact specifically with the enzyme topoisomerase I which relieves torsional strain in DNA by inducing reversible single-strand breaks. Irinotecan and its active metabolite SN-38 bind to the topoisomerase I-DNA complex and prevent religation of these single-strand breaks. Current research suggests that the cytotoxicity of Irinotecan is due to double-strand DNA damage produced during DNA synthesis when replication enzymes interact with the ternary complex formed by topoisomerase I, DNA, and either Irinotecan or SN-38. Mammalian cells cannot efficiently repair these double-strand breaks. The precise contribution of SN-38 to the activity of Irinotecan in humans is not known. Irinotecan is cell cycle phase-specific (S-phase). |
| meticrane |  |  |  |  |
| Hydrastine  hydrochloride |  |  |  |  |
| tyloxapol |  |  | Tyloxapol, when injected IP, blocks plasma lipolytic activity, and thus the breakdown of triglyceride-rich lipoproteins. It has also been shown to be inhibitor of lipoprotein lipase, thus preventing triglyceride uptake. |  |
| Flufenamic acid | Inflammation Pain | anti-proliferativeanti-inflammatory analgesic |  |  |
| sulconazole | Mycoses | anti-fungal |  |  |
| etamsylate |  |  |  |  |
| doxazosin | Hypertension Prostatic Hyperplasia | anti-hypertensive | Doxazosin acts by inhibiting the postsynaptic alpha(1)-adrenoceptors on vascular smooth muscle. This inhibits the vasoconstrictor effect of circulating and locally released catecholamines (epinephrine and norepinephrine), resulting in peripheral vasodilation. | Doxazosin is an alpha-adrenergic blocking agent used to treat hypertension and benign prostatic hyperplasia. Accordingly, Doxazosin is a selective inhibitor of the alpha1 subtype of alpha adrenergic receptors. In the human prostate, Doxazosin antagonizes phenylephrine (alpha1 agonist)-induced contractions, in vitro, and binds with high affinity to the alpha1c adrenoceptor, which is thought to be the predominant functional type in the prostate. Studies in normal human subjects have shown that Doxazosin competitively antagonized the pressor effects of phenylephrine (an alpha1 agonist) and the systolic pressor effect of norepinephrine. The antihypertensive effect of Doxazosin results from a decrease in systemic vascular resistance and the parent compound Doxazosin is primarily responsible for the antihypertensive activity. |
| trioxysalen |  |  |  |  |
| apigenin | Inflammation Neoplasms | anti-inflammatory muscle relaxant (spasmolytic)anti-neoplastic |  |  |
| antimycin_A | Mycoses | anti-fungal |  |  |
| etoposide | Neoplasms | anti-neoplastic | Etoposide inhibits DNA topoisomerase II, thereby inhibiting DNA synthesis at the premitotic stage of cell division. Etoposide is cell cycle dependent and phase specific, affecting mainly the S and G2 phases of cell division. | Etoposide is an antineoplastic agent and an epipodophyllotoxin (a semisynthetic derivative of the podophyllotoxins). It inhibits DNA topoisomerase II, thereby inhibiting DNA synthesis. Etoposide is cell cycle dependent and phase specific, affecting mainly the S and G2 phases. Two different dose-dependent responses are seen. At high concentrations (10 &micro;g/mL or more), lysis of cells entering mitosis is observed. At low concentrations (0.3 to 10 &micro;g/mL), cells are inhibited from entering prophase. It does not interfere with microtubular assembly. The predominant macromolecular effect of etoposide appears to be the induction of DNA strand breaks by an interaction with DNA-topoisomerase II or the formation of free radicals. |
| latamoxef |  |  |  |  |
| Dequalinium  chloride | Bacterial Infections | anti-bacterial |  |  |
| Phthalylsulfat-hiazole | Bacterial Infections | anti-bacterial |  |  |
| repaglinide |  |  | Repaglinide closes ATP-dependent potassium channels in the b-cell membrane by binding at characterizable sites. This potassium channel blockade depolarizes the b-cell, which leads to an opening of calcium channels. The resulting increased calcium influx induces insulin secretion. The ion channel mechanism is highly tissue selective with low affinity for heart and skeletal muscle. | Repaglinide is an oral blood glucose-lowering drug of the meglitinide class used in the management of type 2 diabetes mellitus (also known as non-insulin dependent diabetes mellitus or NIDDM). Repaglinide lowers blood glucose levels by stimulating the release of insulin from the pancreas. This action is dependent upon functioning beta cells in the pancreatic islets. Insulin release is glucose-dependent and diminishes at low glucose concentrations. |
| skimmianine |  |  |  |  |
| atropine | Spasm | muscle relaxant (spasmolytic)smooth muscle relaxant mydriatic antidote | Generally, atropine lowers the "rest and digest" activity of all muscles and glands regulated by the parasympathetic nervous system. This occurs because atropine is a competitive inhibitor of the muscarinic acetylcholine receptors (acetylcholine is the neurotransmitter used by the parasympathetic nervous system). | Atropine, a naturally occurring belladonna alkaloid, is a racemic mixture of equal parts of d- and l-hyoscyamine, whose activity is due almost entirely to the levo isomer of the drug. Atropine is commonly classified as an anticholinergic or antiparasympathetic (parasympatholytic) drug. More precisely, however, it is termed an antimuscarinic agent since it antagonizes the muscarine-like actions of acetylcholine and other choline esters. Adequate doses of atropine abolish various types of reflex vagal cardiac slowing or asystole. The drug also prevents or abolishes bradycardia or asystole produced by injection of choline esters, anticholinesterase agents or other parasympathomimetic drugs, and cardiac arrest produced by stimulation of the vagus. Atropine may also lessen the degree of partial heart block when vagal activity is an etiologic factor. Atropine in clinical doses counteracts the peripheral dilatation and abrupt decrease in blood pressure produced by choline esters. However, when given by itself, atropine does not exert a striking or uniform effect on blood vessels or blood pressure. |
| flunixin |  |  |  |  |
| metyrapone |  |  | The pharmacological effect of Metopirone is to reduce cortisol and corticosterone production by inhibiting the 11-&szlig;-hydroxylation reaction in the adrenal cortex. Removal of the strong inhibitory feedback mechanism exerted by cortisol results in an increase in adrenocorticotropic hormone (ACTH) production by the pituitary. With continued blockade of the enzymatic steps leading to production of cortisol and corticosterone, there is a marked increase in adrenocortical secretion of their immediate precursors, 11-desoxycortisol and desoxycorticosterone, which are weak suppressors of ACTH release, and a corresponding elevation of these steroids in the plasma and of their metabolites in the urine. These metabolites are readily determined by measuring urinary 17-hydroxycorticosteroids (17-OHCS) or 17-ketogenic steroids (17-KGS). Because of these actions, metopirone is used as a diagnostic test, with urinary 17-OHCS measured as an index of pituitary ACTH responsiveness. Metopirone may also suppress biosynthesis of aldosterone, resulting in a mild natriuresis. | Metopirone is an inhibitor of endogenous adrenal corticosteroid synthesis. |
| hydrocotarnine |  |  |  |  |
| thioguanosine |  |  |  |  |
| glibenclamide | Diabetes Mellitus, Type 2 | vasoconstrictor | Sulfonylureas such as glibenclamide likely bind to ATP-sensitive potassium-channel receptors on the pancreatic cell surface, reducing potassium conductance and causing depolarization of the membrane. Depolarization stimulates calcium ion influx through voltage-sensitive calcium channels, raising intracellular concentrations of calcium ions, which induces the secretion, or exocytosis, of insulin. | Glibenclamide (INN), also known as glyburide (USAN), a second-generation sulfonylurea antidiabetic agent, appears to lower the blood glucose acutely by stimulating the release of insulin from the pancreas, an effect dependent upon functioning beta cells in the pancreatic islets. With chronic administration in Type II diabetic patients, the blood glucose lowering effect persists despite a gradual decline in the insulin secretory response to the drug. Extrapancreatic effects may be involved in the mechanism of action of oral sulfonyl-urea hypoglycemic drugs. The combination of glibenclamide and metformin may have a synergistic effect, since both agents act to improve glucose tolerance by different but complementary mechanisms. In addition to its blood glucose lowering actions, glibenclamide produces a mild diuresis by enhancement of renal free water clearance. Glibenclamide is twice as potent as the related second-generation agent glipizide. |
| resveratrol |  |  | Resveratrol suppresses NF-kappaB (NF-kappaB) activation in HSV infected cells. Reports have indicated that HSV activates NF-kappaB during productive infection and this may be an essential aspect of its replication scheme [PMID: 9705914]. | Resveratrol, a phytoalexin, has been found to inhibit herpes simplex virus types 1 and 2 (HSV-1 and HSV-2) replication in a dose-dependent, reversible manner, although this is only one of its many pharmaceutical properties. In some countries where there is higher consumption of red wine, there appears to be a lower incidence of heart disease. Other benefits of resveratrol include its anti-inflammatory and antioxidant effects. In preclinical studies, Resveratrol has been found to have potential anticancer properties. |
| roxithromycin | Bacterial Infections | anti-bacterial immunomodulator | Roxithromycin prevents bacteria from growing, by interfering with their protein synthesis. Roxithromycin binds to the subunit 50S of the bacterial ribosome, and thus inhibits the translocation of peptides. Roxithromycin has similar antimicrobial spectrum as erythromycin, but is more effective against certain gram-negative bacteria, particularly <i>Legionella pneumophila</i>. | Roxithromycin is a semi-synthetic macrolide antibiotic. It is very similar in composition, chemical structure and mechanism of action to erythromycin, azithromycin, or clarithromycin. Roxithromycin has the following antibacterial spectrum <i>in vitro</i>: <i>Streptococcus agalactiae</i>, <i>Streptococcus pneumoniae</i> (Pneumococcus), <i>Neisseria meningitides</i> (Meningococcus), <i>Listeria monocytogenes</i>, <i>Mycoplasma pneumoniae</i>, <i>Chlamydia trachomatis</i>, <i>Ureaplasma urealyticum</i>, <i>Legionella pneumophila</i>, <i>Helicobacter</i> (Campylobacter), <i>Gardnerella vaginalis</i>, <i>Bordetella pertussis</i>, <i>Moraxella catarrhalis</i> (<i>Branhamella Catarrhalis</i>), and <i>Haemophilus ducreyi</i>. Roxithromycin is highly concentrated in polymorphonuclear leukocytes and macrophages, achieving intracellular concentrations greater than those outside the cell. Roxithromycin enhances the adhesive and chemotactic functions of these cells which in the presence of infection produce phagocytosis and bacterial lysis. Roxithromycin also possesses intracellular bactericidal activity. |
| cefalotin | Bacterial Infections | anti-bacterial | The bactericidal activity of cefalotin results from the inhibition of cell wall synthesis via affinity for penicillin-binding proteins (PBPs). | Cefalotin (INN) or cephalothin (USAN) is a semisynthetic first generation cephalosporin having a broad spectrum of antibiotic activity that is administered parenterally. |
| medrysone | Eye Diseases Inflammation | anti-inflammatory | There is no generally accepted explanation for the mechanism of action of ocular corticosteroids. However, corticosteroids are thought to act by the induction of phospholipase A2 inhibitory proteins, collectively called lipocortins. It is postulated that these proteins control the biosynthesis of potent mediators of inflammation such as prostaglandins and leukotrienes by inhibiting the release of their common precursor, arachidonic acid. Arachidonic acid is released from membrane phospholipids by phospholipase A2. | Medrysone is a topical anti-inflammatory corticoidsteroids for ophthalmic use. In patients with increased intraocular pressure and in those susceptible to a rise in intraocular pressure, there is less effect on pressure with medrysone than with dexamethasone or betamethasone. Corticoidsteroids inhibit the edema, fibrin deposition, capillary dilation, and phagocytic migration of the acute inflammatory response, as well as capillary proliferation, deposition of collagen, and scar formation. |
| oxprenolol |  |  | Like other beta-adrenergic antagonists, oxprenolol competes with adrenergic neurotransmitters such as catecholamines for binding at sympathetic receptor sites. Like propranolol and timolol, oxprenolol binds at beta(1)-adrenergic receptors in the heart and vascular smooth muscle, inhibiting the effects of the catecholamines epinephrine and norepinephrine and decreasing heart rate, cardiac output, and systolic and diastolic blood pressure. It also blocks beta-2 adrenergic receptors located in bronchiole smooth muscle, causing vasoconstriction. By binding beta-2 receptors in the juxtaglomerular apparatus, oxprenolol inhibits the production of renin, thereby inhibiting angiotensin II and aldosterone production. Oxprenolol therefore inhibits the vasoconstriction and water retention due to angiotensin II and aldosterone, respectively. | Oxprenolol is a non-selective beta blocker with some intrinsic sympathomimetic activity. Oxprenolol is a lipophilic beta blocker which passes the blood-brain barrier more easily than water soluble beta blockers. As such, it is associated with a higher incidence of CNS-related side effects than hydrophilic ligands such as atenolol, sotalol and nadolol. Oxprenolol is an potent beta-blocker and should not be administered to asthmatics because it can cause irreversible airway failure and inflammation. |
| clindamycin | Bacterial Infections | anti-bacterial | Systemic/Vaginal-Clindamycin inhibits protein synthesis of bacteria by binding to the 50 S ribosomal subunits of the bacteria. Topical-Clindamycin reduces free fatty acid concentrations on the skin and to suppress the growth of Propionibacterium acnes (Corynebacterium acnes) , an anaerobe found in sebaceous glands and follicles. | Clindamycin is an antibiotic, similar to and a derivative of lincomycin. Clindamycin can be used in topical or systemic treatment. It is effective as an anti-anaerobic antibiotic and antiprotozoal. |
| 8-azaguanine |  |  |  |  |

**Annotations and documented effects for the list of compounds whose transcriptional response is similar to that induced by the knock-down of the TNF Network.**

Supplementary Table 1C

| **Drug** | **Known Mode of action** | **Enriched ATC-Codes** | **ATC-Description** | **P-value** | **Enriched Targets** | **p-value** |
| --- | --- | --- | --- | --- | --- | --- |
| apigenin | CDK/TopoII inhibitors | **J01ED** | Long-acting sulfonamides | 6.78E-03 | **SLC6A4** | 4.48E-02 |
| luteolin | CDK/TopoI inhibitors | **J01A** | TETRACYCLINES | 2.28E-02 | *pbpA* | *1.25E-01* |
| chrysin | CDK inhibitors | **J01AA** | Tetracyclines | 2.28E-02 |  | |
| thioguanosine | DNA precursors/antimetabolites | J01E | SULFONAMIDES AND TRIMETHOPRIM | 5.32E-02 |  |  |
| harmine | CDK inhibitors | **J01** | ANTIBACTERIALS FOR SYSTEMIC USE | 1.41E-02 |  |  |
| skimmianine | TopoI inhibitors | **J** | ANTIINFECTIVES FOR SYSTEMIC USE | 1.12E-02 |  |  |
| 0175029-0000 |  | *P* | *ANTIPARASITIC PRODUCTS, INSECTICIDES AND REPELLENTS* | *2.48E-01* |  |  |
| rimexolone |  | *D* | *DERMATOLOGICALS* | *4.16E-01* |  |  |
| sulfametoxydiazine | antibacterials | *S01* | *OPHTHALMOLOGICALS* | *4.16E-01* |  |  |
| trioxysalen |  | *S* | *SENSORY ORGANS* | *4.32E-01* |  |  |
| flunixin |  |  | | |  |  |
| metyrapone |  |  |  |  |  |  |
| cefalotin | antibacterials |  |  |  |  |  |
| irinotecan | TopoI inhibitors |  |  |  |  |  |
| sulfaphenazole | antibacterials |  |  |  |  |  |
| acetylsalicylic_acid |  |  |  |  |  |  |
| metacycline |  |  |  |  |  |  |
| pancuronium_bromide |  |  |  |  |  |  |
| dextromethorphan |  |  |  |  |  |  |
| amoxicillin | antibacterials |  |  |  |  |  |
| lymecycline | antibacterials |  |  |  |  |  |
| 2,6-dimethylpiperidine |  |  |  |  |  |  |
| etidronic_acid |  |  |  |  |  |  |
| ethotoin |  |  |  |  |  |  |
| lysergol |  |  |  |  |  |  |
| vidarabine | antibacterials |  |  |  |  |  |
| Prestwick-664 |  |  |  |  |  |  |
| Prestwick-665 |  |  |  |  |  |  |
| heliotrine |  |  |  |  |  |  |
| todralazine |  |  |  |  |  |  |
| proxymetacaine |  |  |  |  |  |  |
| calcium_pantothenate |  |  |  |  |  |  |
| tiabendazole |  |  |  |  |  |  |
| paroxetine |  |  |  |  |  |  |
| tinidazole | antibacterials |  |  |  |  |  |
| harman | CDK inhibitors |  |  |  |  |  |
| acacetin |  |  |  |  |  |  |
| harmol | CDK inhibitors |  |  |  |  |  |
| nifedipine |  |  |  |  |  |  |

**Enriched drug features for drug community n. 32 in the MANTRA neighbourhood of shCRXCR4**

Supplementary Table 1D

| **Drug** | **Known Mode of action** | **Enriched ATC-Codes** | **ATC-Description** | **P-value** | **Enriched Target** | **p-value** |
| --- | --- | --- | --- | --- | --- | --- |
| DL-thiorphan |  | **S01F** | MYDRIATICS AND CYCLOPLEGICS | 3.93E-03 | **ACHE** | 1.18E-02 |
| atropine | Anticholinergics | **S01FA** | Anticholinergics | 3.93E-03 | **TTR** | 1.18E-02 |
| methacholine_chloride |  | **A03** | DRUGS FOR FUNCTIONAL GASTROINTESTINAL DISORDERS | 4.12E-02 | *CHRM1* | *1.58E-01* |
| papaverine | Anticholinergics | **S01E** | ANTIGLAUCOMA PREPARATIONS AND MIOTICS | 4.12E-02 |  | |
| protoveratrine_A |  | **S01** | OPHTHALMOLOGICALS | 2.41E-02 |  |  |
| labetalol |  | ***S*** | *SENSORY ORGANS* | *2.64E-02* |  |  |
| scopolamine | Anticholinergics | *M01* | *ANTIINFLAMMATORY AND ANTIRHEUMATIC PRODUCTS* | *1.62E-01* |  |  |
| flufenamic_acid |  | *M01A* | *ANTIINFLAMMATORY AND ANTIRHEUMATIC PRODUCTS, NON-STEROIDS* | *1.62E-01* |  |  |
| tremorine | Cholinergic Receptor Agonists | *M* | *MUSCULO-SKELETAL SYSTEM* | *3.36E-01* |  |  |
| bupropion |  | *G* | *GENITO URINARY SYSTEM AND SEX HORMONES* | *3.52E-01* |  |  |
| demeclocycline |  | *A* | *ALIMENTARY TRACT AND METABOLISM* | *3.04E-01* |  |  |
| tomatidine |  | *J01* | *ANTIBACTERIALS FOR SYSTEMIC USE* | *3.59E-01* |  |  |
| hydrastine_hydrochloride |  | *C* | *CARDIOVASCULAR SYSTEM* | *3.57E-01* |  |  |
| clonidine | Anticholinergics | *J* | *ANTIINFECTIVES FOR SYSTEMIC USE* | *4.86E-01* |  |  |
| diclofenac |  | *N* | *NERVOUS SYSTEM* | *5.09E-01* |  |  |
| cefuroxime |  | *D* | *DERMATOLOGICALS* | *5.44E-01* |  |  |
| sulfadiazine |  |  | | |  |  |
| physostigmine | Anticholinergic antagonists |  |  |  |  |  |
| gramine |  |  |  |  |  |  |
| hydroxyachillin |  |  |  |  |  |  |
| ramipril |  |  |  |  |  |  |
| testosterone |  |  |  |  |  |  |
| lidoflazine |  |  |  |  |  |  |
| alpha-yohimbine |  |  |  |  |  |  |
| ifosfamide |  |  |  |  |  |  |

**Enriched drug features for drug community n. 13 in the MANTRA neighbourhood of shCRXCR4**

Supplementary Table 1E

| **Drug** | **Known Mode of action** | **Enriched ATC-Code** | **ATC-Description** | **P-value** | **Enriched Target** | **p-value** |
| --- | --- | --- | --- | --- | --- | --- |
| chlorzoxazone |  | **J01F** | MACROLIDES, LINCOSAMIDES AND STREPTOGRAMINS | 7.89E-03 |  | |
| glibenclamide |  | **A01AB** | Antiinfectives and antiseptics for local oral treatment | 9.57E-03 |  |  |
| clindamycin | antibiotics and bactericidals | **A01** | STOMATOLOGICAL PREPARATIONS | 2.01E-02 |  |  |
| dirithromycin | antibiotics and bactericidals | **A01A** | STOMATOLOGICAL PREPARATIONS | 2.01E-02 |  |  |
| lobeline |  | **S01E** | ANTIGLAUCOMA PREPARATIONS AND MIOTICS | 2.01E-02 |  |  |
| chlortetracycline | antibiotics and bactericidals | *S01A* | *ANTIINFECTIVES* | *6.52E-02* |  |  |
| danazol |  | **S01** | OPHTHALMOLOGICALS | 2.51E-02 |  |  |
| clopamide |  | **S** | SENSORY ORGANS | 2.72E-02 |  |  |
| ajmaline |  | *D* | *DERMATOLOGICALS* | *1.12E-01* |  |  |
| ampyrone |  | *G* | *GENITO URINARY SYSTEM AND SEX HORMONES* | *2.04E-01* |  |  |
| betaxolol |  | *A* | *ALIMENTARY TRACT AND METABOLISM* | *1.38E-01* |  |  |
| chlorhexidine | antibiotics and bactericidals | *J01* | *ANTIBACTERIALS FOR SYSTEMIC USE* | *1.70E-01* |  |  |
| methazolamide |  | *J* | *ANTIINFECTIVES FOR SYSTEMIC USE* | *2.52E-01* |  |  |
| hydrastinine |  | *C* | *CARDIOVASCULAR SYSTEM* | *3.45E-01* |  |  |
| Prestwick-689 |  | **J01F** | MACROLIDES, LINCOSAMIDES AND STREPTOGRAMINS | 7.89E-03 |  |  |
| acetylsalicylic_acid |  | **A01AB** | Antiinfectives and antiseptics for local oral treatment | 9.57E-03 |  |  |
| metacycline |  |  | | |  |  |
| pancuronium_bromide |  |  |  |  |  |  |
| dextromethorphan |  |  |  |  |  |  |
| amoxicillin | antibacterials |  |  |  |  |  |
| lymecycline | antibacterials |  |  |  |  |  |
| 2,6-dimethylpiperidine |  |  |  |  |  |  |
| etidronic_acid |  |  |  |  |  |  |
| ethotoin |  |  |  |  |  |  |
| lysergol |  |  |  |  |  |  |
| vidarabine | antibacterials |  |  |  |  |  |
| Prestwick-664 |  |  |  |  |  |  |
| Prestwick-665 |  |  |  |  |  |  |
| heliotrine |  |  |  |  |  |  |
| todralazine |  |  |  |  |  |  |
| proxymetacaine |  |  |  |  |  |  |
| calcium_pantothenate |  |  |  |  |  |  |
| tiabendazole |  |  |  |  |  |  |
| paroxetine |  |  |  |  |  |  |
| tinidazole | antibacterials |  |  |  |  |  |
| harman | CDK inhibitors |  |  |  |  |  |
| acacetin |  |  |  |  |  |  |
| harmol | CDK inhibitors |  |  |  |  |  |
| nifedipine |  |  |  |  |  |  |

**Enriched drug features for drug community n. 42 in the MANTRA neighbourhood of shCRXCR4**

Supplementary Table 1F

| **Drug** | **Known Mode of action** | **Enriched ATC-Code** | **ATC-Description** | **P-value** | **Enriched Target** | **p-value** |
| --- | --- | --- | --- | --- | --- | --- |
| sanguinarine | Anticoagulant | **B02B** | VITAMIN K AND OTHER HEMOSTATICS | 4.62E-04 | **PDE4A** | 6.32E-03 |
| cantharidin |  | **B02** | ANTIHEMORRHAGICS | 2.70E-03 | **ADRA2A** | 2.59E-02 |
| 8-azaguanine |  | **B** | BLOOD AND BLOOD FORMING ORGANS | 7.02E-03 |  |  |
| verteporfin |  | *R03* | *DRUGS FOR OBSTRUCTIVE AIRWAY DISEASES* | *8.22E-02* |  |  |
| ginkgolide_A | Anticoagulant | *G* | *GENITO URINARY SYSTEM AND SEX HORMONES* | *3.81E-01* |  |  |
| talampicillin |  | *J01* | *ANTIBACTERIALS FOR SYSTEMIC USE* | *3.98E-01* |  |  |
| menadione | Hemostatic agents | *R* | *RESPIRATORY SYSTEM* | *5.22E-01* |  |  |
| ipratropium_bromide |  | *J* | *ANTIINFECTIVES FOR SYSTEMIC USE* | *5.29E-01* |  |  |
| hydrocotarnine |  | *S01* | *OPHTHALMOLOGICALS* | *5.78E-01* |  |  |
| dacarbazine |  | *S* | *SENSORY ORGANS* | *5.92E-01* |  |  |
| etamsylate | Hemostatic agents |  |  |  |  |  |
| solanine |  |  |  |  |  |  |
| dipyridamole | Hemostatic agents |  |  |  |  |  |
| N-acetyl-L-leucine |  |  |  |  |  |  |
| desoxycortone |  |  |  |  |  |  |
| epivincamine |  |  |  |  |  |  |
| zimeldine |  |  |  |  |  |  |
| tracazolate | modulation of GABA receptor |  |  |  |  |  |
| pargyline | Antihypertensive agents |  |  |  |  |  |
| sitosterol | Antihypertensive agents |  |  |  |  |  |
| picrotoxinin | modulation of GABA receptor |  |  |  |  |  |
| 6-benzylaminopurine |  |  |  |  |  |  |
| altizide | Antihypertensive agents |  |  |  |  |  |
| terbutaline |  |  |  |  |  |  |
| ketoprofen | Anticoagulant |  |  |  |  |  |
| laudanosine | modulation of GABA receptor |  |  |  |  |  |
| phentolamine | Antihypertensive agents |  |  |  |  |  |
| tolbutamide |  |  |  |  |  |  |
| flumequine |  |  |  |  |  |  |
| oxytetracycline |  |  |  |  |  |  |
| sotalol | Antihypertensive agents |  |  |  |  |  |
| methyldopa | Antihypertensive agents |  |  |  |  |  |
| R-atenolol | Antihypertensive agents |  |  |  |  |  |

**Enriched drug features for drug community n. 89 in the MANTRA neighbourhood of shCRXCR4**
